# The complete and closed genome of the facultative generalist *Candidatus* Endoriftia persephone from deep‐sea hydrothermal vents

**DOI:** 10.1111/1755-0998.13668

**Published:** 2022-06-30

**Authors:** André Luiz De Oliveira, Abhishek Srivastava, Salvador Espada‐Hinojosa, Monika Bright

**Affiliations:** ^1^ Department of Functional and Evolutionary Ecology University of Vienna Vienna Austria

**Keywords:** endosymbiont, evolutionary theory, genomics, long‐read sequencing, molecular evolution, *Riftia pachyptila*

## Abstract

The mutualistic interactions between *Riftia pachyptila* and its endosymbiont *Candidatus* Endoriftia persephone (short Endoriftia) have been extensively researched. However, the closed Endoriftia genome is still lacking. Here, by employing single‐molecule real‐time sequencing we present the closed chromosomal sequence of Endoriftia. In contrast to theoretical predictions of enlarged and mobile genetic element‐rich genomes related to facultative endosymbionts, the closed Endoriftia genome is streamlined with fewer than expected coding sequence regions, insertion‐, prophage‐sequences and transposase‐coding sequences. Automated and manually curated functional analyses indicated that Endoriftia is more versatile regarding sulphur metabolism than previously reported. We identified the presence of two identical rRNA operons and two long CRISPR regions in the closed genome. Additionally, pangenome analyses revealed the presence of three types of secretion systems (II, IV and VI) in the different Endoriftia populations indicating lineage‐specific adaptations. The *in depth* mobilome characterization identified the presence of shared genomic islands in the different Endoriftia drafts and in the closed genome, suggesting that the acquisition of foreign DNA predates the geographical dispersal of the different endosymbiont populations. Finally, we found no evidence of epigenetic regulation in Endoriftia, as revealed by gene screenings and absence of methylated modified base motifs in the genome. As a matter of fact, the restriction‐modification system seems to be dysfunctional in Endoriftia, pointing to a higher importance of molecular memory‐based immunity against phages via spacer incorporation into CRISPR system. The Endoriftia genome is the first closed tubeworm endosymbiont to date and will be valuable for future gene oriented and evolutionary comparative studies.

## 1 INTRODUCTION

Many symbiotic mutualisms involve horizontally transmitted microbes that live solitary in the environment as well as sheltered in/on eukaryote hosts (Bright & Bulgheresi, 2010; Douglas, 2010). Often for both symbiont and host the mutualism is facultative. Mutualism for the giant tubeworm *Riftia pachyptila* (short *Riftia*), however, is strictly obligate (Bright & Lallier, 2010). High dependency on its sole partner *Candidatus* Endoriftia persephone (short Endoriftia) is apparent already in *Riftia*'s (and other vestimentiferan) aposymbiotic larvae that must pick up the suitable partner from the hydrothermal vent environment once they settle (Nussbaumer et al., 2006). Later, the gutless adult only survives if the provision of inorganic nutrients for the chemotrophic sulphur oxidizing Endoriftia is reciprocated with released organic carbon (Bright et al., 2000; Childress & Girguis, 2011; Felbeck & Jarchow, 1998). In addition, the host also digests the bacteria (Bosch & Grassé, 1984; Bright & Sorgo, 2003; de Oliveira et al., 2021; Hand, 1987; Hinzke et al., 2019, 2021). In contrast, apparently, life for host‐associated Endoriftia is facultative as evidenced by the uptake of these bacteria during horizontal transmission (Nussbaumer et al., 2006) from a free‐living environmental pool detected at East Pacific Rise hydrothermal vents and adjacent cold deep‐sea basalts and sediments as well as at the pelagic zone (Harmer et al., 2008; Polzin et al., 2019). Further, release of symbionts upon host death was shown experimentally to lead to free‐living dividing populations in high pressure vessels (Klose et al., 2015), indicative of proliferating free‐living Endoriftia in the environment. Several ribosomal and nuclear marker studies showed that Endoriftia is present in different vestimentiferans other than *Riftia* (i.e., *Tevnia jerichonana, Ridgeia piscesae*, *Oasisia alvinae, Esparpia spicata* at vents only) ruling out the codiversification of this bacterium with their respective hosts (Di Meo et al., 2000; Feldman et al., 1997; Nelson & Fisher, 2000).

Theory establishes that partner choice is a mechanism for positive assortment between cooperating partners (Fletcher & Doebeli, 2009; Queller, 1985) that acts prior establishment of the association (Bull & Rice, 1991). In horizontally transmitted symbionts, partner choice is expected to ensure the selection of the cooperating partner. This mechanism is selective enough not to allow any other microbe to enter but is also permissive enough to allow polyclonal symbiont populations selected from the environment to establish in the host (Genkai‐Kato & Yamamura, 1999; Heath & Stinchcombe, 2014; Vrijenhoek, 2010). Indeed, many different associations involving horizontal transmitted symbionts house polyclonal symbiont populations which are selected from the environment (Perez et al., 2021; Polzin et al., 2019; Sachs et al., 2009; Wollenberg & Ruby, 2009). Unique among horizontal transmitted symbionts, however, is that *Riftia* and other vestimentiferan hosts have a single symbiont‐housing organ, the trophosome, in which a single strain of Endoriftia dominates over many others, as evidenced by multilocus gene sequencing (Perez et al., 2021; Polzin et al., 2019) and metagenomics (Polzin et al., 2019). This is in contrast to other horizontally transmitted symbioses as those involving the bobtail squid (Wollenberg & Ruby, 2009) or the legumes (Sachs et al., 2009) in which several symbiont‐housing organ compartments are present with each harbouring usually one or two strains. In this respect, it is important to stress that studies on the genetic diversity of Endoriftia are focused on small discreet parts of the trophosome of single individual hosts, thus not providing a complete view of the genetic makeup of *Riftia*'s endosymbionts across the entirety of the large symbiont‐housing organ nor across the *Riftia* population level.

The first draft genome of this facultative generalist bacterium was obtained from *Riftia* (Robidart et al., 2008), followed by further draft genomes from *Riftia* and the other vent host species *Tevnia jerichonana* and *Ridgeia piscesae* (Gardebrecht et al., 2012; Perez & Juniper, 2016). However*,* so far, despite extensive in silico and experimental investigations, a complete and closed Endoriftia genome is still lacking. The varying degrees of the Endoriftia genome fragmentation affect many “genome‐centric” analyses (e.g. gene content investigations, synteny, pangenome analyses) and negatively impact gene prediction and annotation (e.g. false positive/negative annotation rates (Klassen & Currie, 2012)), thus hampering downstream analyses and the correct biological interpretation of the data. Here, by using accurate circular consensus long‐read sequencing (Wenger et al., 2019), we closed the genome of the dominant strain of *Riftia*'s endosymbiont *Ca.* Endoriftia persephone. Moreover, we thoroughly characterized, in a broad comparative evolutionary genomics framework, the Endoriftia genome in relation to its metabolic capabilities, defence system mechanisms, genetic mobile elements, and epigenetic regulation. To date, the Endoriftia genome constitutes the first closed tubeworm endosymbiont's genome publicly available and will be an important resource to broaden our understanding not only about *Riftia*‐Endoriftia symbiosis, but also for other chemosynthetic animal‐microbe systems.

## 2 MATERIALS AND METHODS

### 2.1 Sample collection and sequencing

A sample of trophosome obtained from a female *Riftia pachyptila* collected at the Tica hydrothermal vent site (Alvin dive 4839, 9° 50.398 N, 104° 17.506 W, 2514 m depth, 2016) was separated from the host tissue as described in Polzin et al. (2019). The DNA of the enriched endosymbiont fraction was extracted using the Qiagen Blood & Cell Culture DNA Mini Kit (cat no./ID: 13323) following the manufacturer's instructions. The long‐read metagenome library was prepared using the PacBio large‐insert DNA protocol and the SMRTbell Express Kit. The sequencing was performed on a Sequel1. The library construction and sequencing were performed at the Vienna BioCenter Core Facilities (https://www.viennabiocenter.org/vbcf/). Highly accurate single‐molecule consensus reads (HiFi reads) with a minimum of three passes were generated from the raw sequence data using the ccs tool present in the “PacBio Secondary Analysis Tools on Bioconda”. The HiFi reads were assembled with Flye version 2.5 (Kolmogorov et al., 2019). The circularized contig containing the full genome of Endoriftia was polished using the tool arrow available at the “PacBio Secondary Analysis Tools” on Bioconda package. Genome completeness was performed with the CheckM software using the “Gammaproteobacteria” lineage (Parks et al., 2015). We manually fixed the first codon of the gene *dnaA* as the chromosome origin in the closed, already circularized, and polished Endoriftia genome.

### 2.2 Annotation and genotyping

To annotate the genome we employed a nested approach of integrative levels (Novikoff, 1945). The predicted genes were treated as a part of identifiable metabolic pathways and these were grouped as functional traits, which are understood as microbial characteristics linked to the microbe's fitness (Green et al., 2008). Other relevant structural features were also treated as traits (e.g. transporters, storage capabilities). Additionally, we further annotated the protein coding sequences predicted from the closed Endoriftia genome using automated tools such as Prokka version 1.14.0 (Seemann, 2014), eggNOG mapper version 2 (Huerta‐Cepas et al., 2017, 2019), and KEGG through the web‐based server KAAS (Moriya et al., 2007) followed by manual curation of the automatic results. The identification of the mobile genetic elements (i.e., mobilome) was performed with the online version of ISfinder (https://isfinder.biotoul.fr/) (Kichenaradja et al., 2010; Siguier et al., 2006), IslandViewer4 (https://pathogenomics.sfu.ca/islandviewer) (Bertelli et al., 2017), alien hunter (https://www.sanger.ac.uk/tool/alien‐hunter/), PHASTER (https://phaster.ca/) (Arndt et al., 2016; Zhou et al., 2011), SpacePHARER (Zhang et al., 2021), and BacANT (Hua et al., 2021). CRISPR regions were further investigated and characterized with CRISPRCasFinder browser (https://crisprcas.i2bc.paris‐saclay.fr/CrisprCasFinder/Index) (Couvin et al., 2018). To enrich the comparative genomic analyses we downloaded, filtered, and assembled the host‐associated metagenome described in Polzin et al. (2019), using BBDuk version38.42 and metaSPAdes version 3.13.0 (Nurk et al., 2017), respectively. Genotyping was performed by comparing four housekeeping loci retrieved from the closed genome, (i.e., *atpA*, *gyrB*, *recA* and *uvrD*) against the sequence types generated by Polzin et al. (2019) through BLASTN similarity searches (Camacho et al., 2009).

### 2.3 Methylome analyses

The base modification analysis was performed with SMRTLink version 9.0.0.92188, and the screening of important methyltransferase enzymes was executed with hmmsearch version 3.2.1 using MTases PFAM domains (PF00145, P02384, PF01555).

### 2.4 Comparative genomic analyses

Average nucleotide identity index analysis was performed with OAU (OrthoANI) tool (Lee et al., 2016) and publicly available tubeworm endosymbiont genomes. Three orthology analysis inferences (*phylogenomic set* (*N* = 13): all Endoriftia genomes, other available tubeworm endosymbionts and *Sedimenticola thiotaurini*; *pangenome set* (*N* = 7): solely Endoriftia genomes; and, *positive selection set* (*N* = 7): the complete closed Endoriftia genome, all available non‐Endoriftia tubeworm endosymbionts, and *Sedimenticola thiotaurini*) were performed with Orthofinder version 2.3.8 (Emms & Kelly, 2019).

The phylogenomic analysis was performed with a super‐matrix generated with FasConCAT version 1 (Kück & Meusemann, 2010) by concatenating the single copy orthologue sequences present in the *phylogenomic set*. The phylogenomic tree inference was performed on a partition model analysis using IQ‐TREE version 1.6.11 combining ModelFinder, tree search, and 1000 ultra‐fast bootstrap (Hoang et al., 2018; Kalyaanamoorthy et al., 2017 ; Nguyen et al., 2015). The Venn diagrams were obtained using the package venn available at R (https://CRAN.R‐project.org/package=venn). Circular plots were generated using Circos (Krzywinski et al., 2009).

The pangenome (persistent, shell, cloud) was calculated with the tool PPanGGOLiN version 1.1.136 (Gautreau et al., 2020) using the six previously published Endoriftia draft genomes (Gardebrecht et al., 2012; Perez & Juniper, 2016; Polzin et al., 2019; Robidart et al., 2008) and the closed genome herein reported. The Orthofinder results obtained from the *pangenome set* were used as input in PPanGGOLiN version 1.1.136 under the “‐‐clusters” parameter.

Nonsynonymous (dN) and synonymous (dS) substitution rates in the closed Endoriftia genome were calculated with PAML/CODEML version 4.8a package (Yang, 2007) using the Orthofinder version 2.3.8 *positive selection set* results. Briefly, the single copy protein orthologues identified in Orthofinder version 2.3.8 and the corresponding gene sequences were converted into a codon alignment using the software PAL2NAL (Suyama et al., 2006). Gene trees were inferred from codon alignments with IQ‐TREE version 1.6.11 (Kalyaanamoorthy et al., 2017; Nguyen et al., 2015) and used in the subsequent analyses. Pairwise estimates of dN/dS ratio obtained from positive (model = 2, NSsites = 2, fix_omega = 0, omega = 1) and null (model = 2, NSsites = 2, fix_omega = 1, omega = 1) branch‐site models (Zhang et al., 2005) specific to the Endoriftia closed genome lineage were calculated using PAML/CODEML version 4.8a package. The closed Endoriftia lineage was defined as “foreground branch” and assumed to contain different values of dN/dS, whereas the dN/dS of all other branches of the tree (i.e., “background” branches) contained a fixed distribution. The estimated log likelihoods of the positive and null models were then used to construct likelihood ratio tests and the *p*‐values were obtained employing the following formula: *p*‐value = *χ* ^2^ (2*ΔlnL, degree of freedom); where the degree of freedom is 1. The aforementioned pipeline was performed one more time with the single copy orthologues obtained from the *phylogenomic set*. Since recombination events can falsely be interpreted as signs of molecular adaption (Anisimova et al., 2003), we identified positive selected genes which were located in inferred recombination hotspots in the closed Endoriftia genome. Recombination events were inferred using ClonalFrameML (Didelot & Wilson, 2015) by specifying a starting phylogenetic tree and the core alignment blocks (>500 nt) extracted from the multiple sequence alignment generated with progressiveMauve version 2.4.0 (Darling et al., 2010), and the genomes present in the *positive selection set*. All previous bioinformatic tools were executed with the default parameters.

The positively selected genes present in the nonrecombinant regions of the closed Endoriftia genome were annotated with InterproScan version 5.39–77.0 (Jones et al., 2014) and the enrichment analysis for GO was performed with topGO version 2.36.0 (https://bioconductor.org/packages/release/bioc/html/topGO.html) using Fisher's exact test against the Endoriftia background (all genes identified in the closed Endoriftia genome) coupled with weight01 algorithm (Alexa et al., 2006).

## 3 RESULTS AND DISCUSSION

### 3.1 Overview of the closed genome of *Ca.* Endoriftia persephone

**FIGURE 1:**
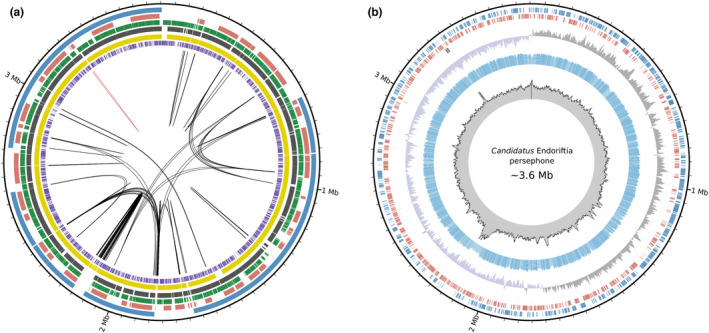
Overview of the complete and closed genome of Endoriftia and publicly available drafts. (a) The ~3.6 Mb closed genome of *Candidatus* Endoriftia persephone. The six distinct publicly available Endoriftia drafts were mapped back to the closed genome presented herein. The concentrical circles from the centre to the periphery represent in purple (Robidart et al., 2008); in yellow and black (Gardebrecht et al., 2012); in green and maroon (Perez & Juniper, 2016) and in cyan (Polzin et al., 2019) the different draft metagenomes. The black and red lines in the middle of the figure represent repetitive regions >5 kpb and >500 pb in the endosymbiont closed genome, respectively. (b) Circos plot showing the PacBio coverage across the closed genome (grey histograms; ~2000× coverage). The GC content is represented by the blue histograms. GC skew data is shown by the grey and purple histograms. Blue and red boxes correspond to coding sequence regions in the forward and reverse strands of the closed Endoriftia genome, respectively. The two grey boxes correspond to the rRNA clusters in the closed *Ca.* Endoriftia genome

**FIGURE 2:**
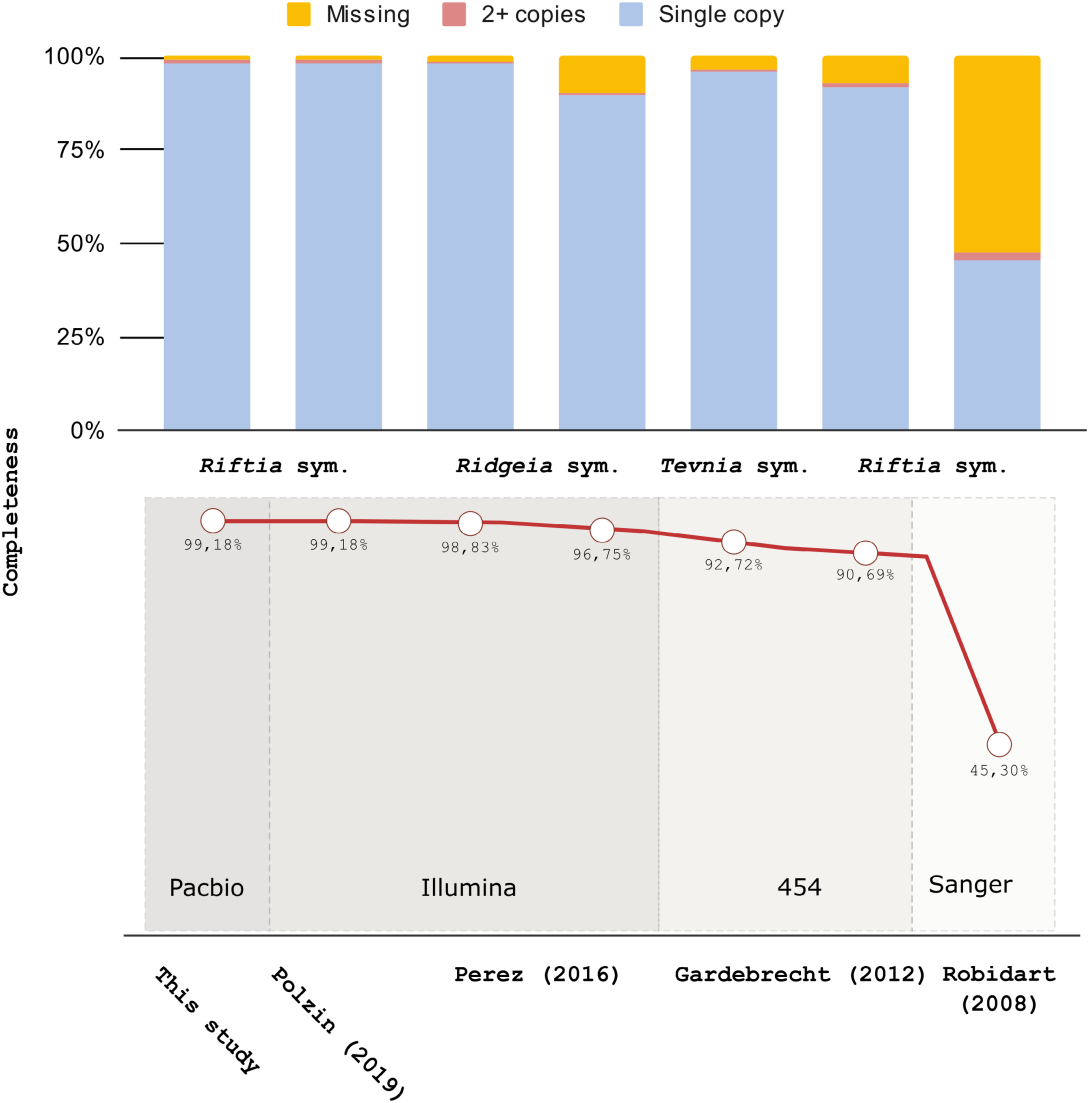
The completeness of the closed *Candidatus* Endoriftia persephone genome and publicly available drafts. The stacked proportional bar charts reflect the number of missing, multicopies, and single copy genes in the different genome drafts of Endoriftia as shown by CheckM analysis

The draft (meta)genome of Endoriftia was first generated by Robidart et al. (2008) using Sanger sequencing. Further pyrosequencing, short‐read sequencing and genome assemblies of Endoriftia greatly improved contiguity (Gardebrecht et al., 2012; Perez & Juniper, 2016), however, a closed genome was not obtained (Figure 1a). Using accurate single‐molecule consensus reads (PacBio HiFi reads) we sequenced and closed with ~2000 fold‐coverage (~120× circular consensus sequencing with three passes) the Endoriftia genome (Figure 1b). The genome size is ~3.6 Mb and presents a completeness of 99.18%, as revealed by CheckM (Parks et al., 2015), rendering this genome the first closed and most complete Endoriftia genome (Figure 2) (Gardebrecht et al., 2012; Perez & Juniper, 2016; Polzin et al., 2019; Robidart et al., 2008) and of a vestimentiferan endosymbiont to date (Breusing et al., 2020; Gardebrecht et al., 2012; Li et al., 2018, 2019; Robidart et al., 2008). The extremely low strain homogeneity and contamination levels (0% and 1.05%, respectively; Supplementary Table S1) are a clear indicative that the complete closed Endoriftia genome is not composed of closely related strains (i.e., chimeric assembly). Furthermore, the sequences of the dominant strain of *Riftia*‐associated Endoriftia present at the vent site Tica at the East Pacific Rise, generated by Polzin et al. (2019) and the respective orthologs in the complete closed genome (also sampled in Tica) were identical, showing that the genome herein described belongs to the dominant strain of Endoriftia.

**FIGURE 3:**
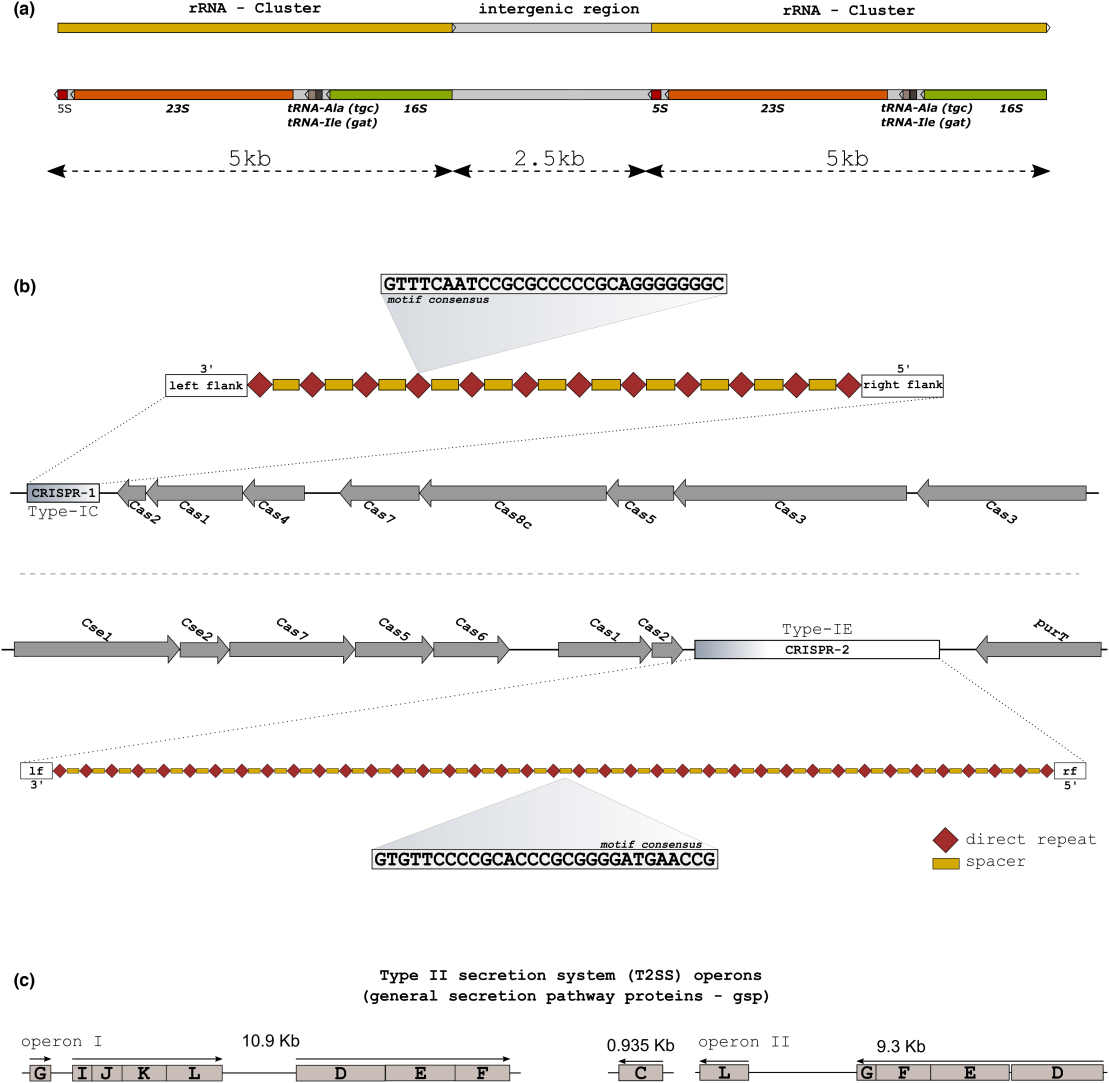
Genomic features obtained from the closed Endoriftia genome. (a) Two identical rRNA operons located in the closed Endoriftia genome. (b) Two large CRISPR (clustered regularly interspaced short palindromic repeat) regions composed of 12 and 39 direct repeats units, with respective sizes of ~9.4‐ and ~11.6 k present in the closed Endoriftia genome. (c) Type II secretion system (T2SS) operons in the closed Endoriftia genome

The genome encodes 3217 coding sequences (CDS), from which 319 present a signal peptide sequence, 49 tRNAs coding for all 20 amino acids, 12 non‐coding RNAs and six rRNA genes (two copies of 5S, 16S and 23S) (Table 1) located in two distinct and identical chromosomal clusters, contradicting previous studies (Perez & Juniper, 2016; Polzin et al., 2019) (Figure 3a). Interestingly, two tandem rRNA gene clusters with the exact syntenic organization (including the intergenic tRNAs) were previously detected in a *Ridgeia*‐associated raw metagenomic assembly (JGI's IMG‐Mer database; GOLD analysis ID project: Ga0041824; contig Draft11:123922) (Perez & Juniper, 2016). Consistent with earlier studies (Perez et al., 2021; Perez & Juniper, 2016), but contradicting Gardebrecht et al. (2012) (*Riftia1* draft genome), we recovered two large and complete CRISPR (clustered regularly interspaced short palindromic repeat) regions composed of 12 and 39 direct repeats units, with respective sizes of ~9.4‐ and ~11.6‐kb (Figure 3b). The two clusters belong to class I, type C and –E, respectively, with the conserved CRISPR2 region located ~370 bp upstream of the gene *purT* (phosphoribosylglycinamide formyltransferase 2), as recently described by Perez et al. (2021). It has been shown in a recent study (Perez et al., 2021) that CRISPR regions present in *Ridgeia*‐associated Endoriftia contain a higher genetic diversity than housekeeping genes. In our analyses, we did not observe in the closed Endoriftia genome any intragenomic conservation of spacer sequences (Data S1), pointing as well to a high genetic variability of these spacer sequences. Since the dynamics and evolution of bacterial adaptive immunity are shaped by context‐dependent environmental factors (e.g. local virome) and different bacterial strains often harbour distinct CRISPR loci (Westra et al., 2016), CRISPR‐based genotyping could improve the final strain‐level characterization of uncultured *Riftia*‐associated (and free‐living) Endoriftia populations.

**TABLE 1:** Overview of the publicly available Endoriftia metagenomes and the complete and closed genome based on Prokka version 1.14.0 annotation (Seemann, 2014)

Assembly name	Number of contigs	Total length	Largest contig	GC (%)	N_50_	Gene	CDS	rRNA	Repeat region	tRNA	tmRNA
This_study	1	3,604,761	3,604,761	58.59	3,604,761	3282	3217	6	2	49	1
*Riftia* sym. (Polzin et al., 2019)	12	3,534,855	1,660,857	58.77	531,644	3262	3197	6	2	49	1
*Ridgeia* sym. 1 (Perez & Juniper, 2016)	97	3,441,033	236,298	58.88	83,967	3223	3162	3	1	47	1
*Ridgeia* sym. 2 (Perez & Juniper, 2016)	693	3,417,427	40,802	58.87	7630	3485	3428	3	0	43	1
*Tevnia* sym. (Gardebrecht et al., 2012)	184	3,637,102	248,702	58.16	92,701	3447	3389	4	4	44	1
*Riftia* sym. (Gardebrecht et al., 2012)	197	3,481,040	95,370	58.82	29,688	3462	3407	3	4	42	1
*Riftia* sym. (Robidart et al., 2008)	2170	3,203,254	10,451	57.87	1953	4845	4805	3	3	30	1

Functional annotation of the closed Endoriftia genome using eggnog‐mapper version 2 revealed that 2899 predicted genes (~89.9%) could be assigned to 20 distinct broad cluster of orthologous genes (COG) categories, with the categories “function unknown (S)”, “energy production and conversion (C)” and “signal transduction mechanisms (T)” containing the greatest number of genes (Table S1). The percentage of uncharacterised predicted genes (i.e., genes with either unknown function or that remain unannotated) in Endoriftia genome (797 genes/25% of the total) and in the most complete genome of cold‐seep endosymbiont available to date (endosymbiont of *Lamellibrachia barhami*; 1239/27%) (Breusing et al., 2020) is similar. This indicates that the function of a large number of genes in the tubeworm's endosymbiont genomes remains unknown.

After detecting positive selection and identifying particular changes in patterns of amino acid conservation (i.e., signs of positive selection at a protein/codon level), we identified a small percentage of genes in the Endoriftia closed genome showing signatures of adaptative evolution (*p*‐value < 0.05; 223 genes /6.93%) in comparison to cold‐seep tubeworm endosymbionts (*positive selection set*; *N* = 7) (*Lamellibrachia*, *Paraescarpia, Seepiophila)* and the free‐living closely related bacterium *Sedimenticola* (Table S1). The positively selected genes are involved in a wide range of biological activity including cell cycle (e.g. *ftsZ*, *ftsY*, *ftsK*, *flgD*, *fliK*), DNA replication/repair (e.g. *holA*, *uvrA, parE, gyrA, smc2*), stress response (e.g. *lepA*, *hscB*), and cofactor biosynthesis (e.g. *moaB, btuF*, *hemF*, *hemY*). Furthermore, gene ontology (GO) analyses performed on the positively selected genes show an enrichment of distinct molecular function terms related to binding (GTP, folic acid, and purine ribonucleoside), aminoacyl‐tRNA hydrolase, ATP hydrolysis and peptidase activities (Figure S1). Due to the low nucleotide sequence divergence among the different Endoriftia populations within mid‐ocean ridge systems (Perez & Juniper, 2016), only two genes with signs of positive selection were identified in the complete closed genome in comparison to cold‐seep tubeworm endosymbionts (*Lamellibrachia*, *Paraescarpia, Seepiophila),* the free‐living closely related bacterium *Sedimenticola thiotaurini* and the different Endoriftia draft genomes (*phylogenomic set*; *N* = 13; Table S1). These genes, *mreC* (cell shape‐determining protein) and *hemB* (delta‐aminolaevulinic acid dehydratase)*,* are involved in cell division/shape (van den Ent et al., 2006) and haem biosynthesis (Choby & Skaar, 2016), respectively. Although more adequate metrics exist to detect positive selection in closely related bacterial populations (pN/pS), the previous results might indicate sympatric adaptations in the complete closed genome herein described and other Endoriftia populations (Table S1).

### 3.2 Metabolic capabilities of Endoriftia

**FIGURE 4:**
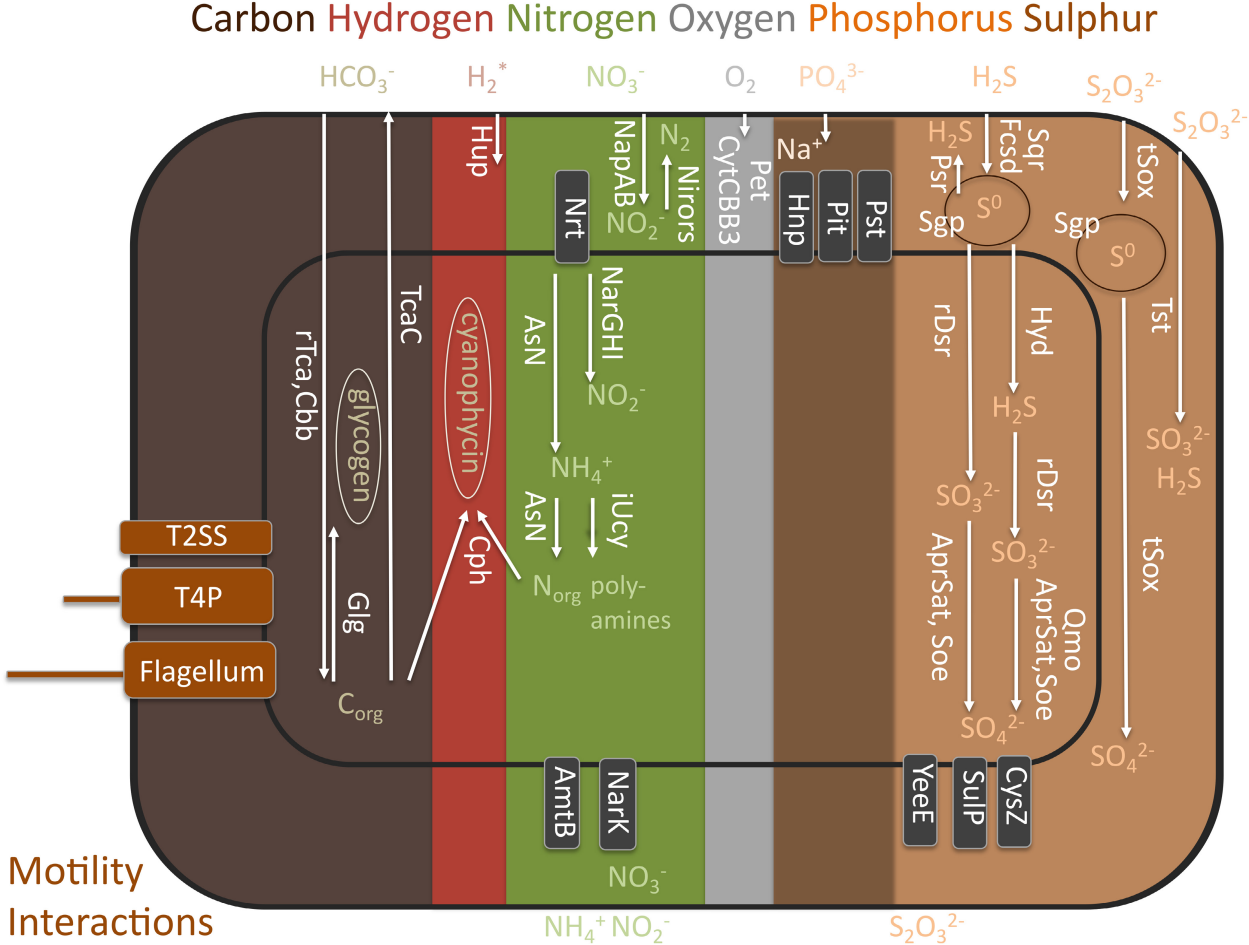
Major metabolic capabilities found in the closed Endoriftia genome. Selected relevant potential functional capabilities of *Candidatus* Endoriftia persephone inferred from its closed genome are shown (for simplicity not all transporters are depicted in the figure). Predicted and functionally annotated genes are grouped in metabolic pathways. Functional traits are features such as single metabolic pathways or composites of them that affect the organism fitness. Structural features such as transporters or secretion systems, and storage capabilities are also considered as traits. Trait labels are generally in white fonts and in vertical orientation, except for the motility and interactions on the left. Compound labels are horizontally oriented. Storage compartments are indicated with ellipses. AmtB, amonium transporter; AprSat, sulphate adenylyltransferase and adenylylsulphate reductase sulphite oxidation; AsN, assimilatory nitrate reduction; Cbb, Calvin‐Benson‐Basham cycle; Cph, cyanophycin biosynthesis; CysZ, sulphate transporter; CytCBB3, cytochrome cbb_3_ based oxygen respiration; Fcsd, flavocytochrome c sulphide dehydrogenase sulphide oxidation; Glg, glycogen biosynthesis; Hnp, high affinity sodium‐phosphate symporter; Hup*, putative hydrogen oxidation (Mitchell et al., 2019 found uptake of hydrogen apparently does not serve to fuel carbon fixation, neither was hydrogenase activity measured); Hyd, sulhydrogenase elemental sulphur oxidation; iUcy, incomplete urea cycle lacking last step arginase gene; NapAB, periplasmic nitrate reduction to nitrite; NarGHI, cytoplasmic nitrate reduction to nitrite; NarK, nitrate‐nitrite antiporter; Nirors, sequential oxidation from nitrate to dinitrogen by nirS, norBC and nosZ as final steps of the denitrification process; Nrt, nitrate transporter; Pet, cytochrome bc_1_ complex mediated electron transport chain; Pit, low affinity phosphate transporter; Psr, polysulphide reductase elemental sulphur reduction; Pst, high affinity phosphate transporter; Qmo, quinone‐interacting membrane‐bound oxidoreductase mediated sulphite oxidation; rDsr, reverse dissimilatory sulphate reductase mediated sulphur oxidation; rTca, reductive TCA cycle; Sgp, sulphur globule proteins; Soe, sulphite‐oxidation enzyme sulphite oxidation; Sqr, sulphide:quinone oxydoreductase sulphide oxidation; SulP, sulphate permease; T2SS, type II secretion system; T4P, type IV pilus; TcaC, TCA cycle; tSox, truncated Sox mediated sulphur oxidation; Tst, thiosulphate disproportionation; YeeE, thiosulphate transporter

To further explore the potential metabolic capabilities of Endoriftia we combined multiple functional annotation tools and performed an exhaustive manual curation (Table S2). Our results corroborate most previous findings of genes involved in autotrophy, N metabolism, sulphur oxidation and motility (Gardebrecht et al., 2012; Kleiner et al., 2012; Markert et al., 2007; Perez & Juniper, 2016; Robidart et al., 2008) (Figure 4), in addition to novel genes and potential new metabolic capabilities in Endoriftia. Genes related to two carbon‐fixation pathways were present in the genome, the Calvin‐Benson‐Bassham cycle (Cbb) and the reductive TCA cycle (rTca) (Felbeck, 1985; Hinzke et al., 2019; Minic & Hervé, 2004). Cbb is performed by a form II RuBisCO (Badger & Bek, 2008) and no carboxysome genes were found (Scott et al., 1999). As previously described, a CO_2_ concentration mechanism was indicated by the presence of carbonic anhydrase genes belonging to the alpha and beta classes (Robidart et al., 2008). We, additionally, identified another carbonic anhydrase gene in the closed Endoriftia that falls into the gamma class (discussed more in details below). The reductive TCA cycle is supported by the presence of the two genes *aclA* and *aclB* (Leonard et al., 2021), based in the conserved domain composition of *aclA* (Nunoura et al., 2018), and the gene cluster synteny with *Thioflavicoccus mobilis* (Rubin‐Blum et al., 2019) and phylogenetic inferences (Figure S2). A contiguous gene cluster *korDABC* encoding for the tetrameric version of 2‐oxoglutarate:ferrodoxin oxidoreductase was also found upstream, and in the vicinity, of other genes involved in the reductive TCA cycle (Rubin‐Blum et al., 2019). Two copies of dimeric versions *korAB* are also present in the genome and are thought to be capable of acting in both oxidative and reductive directions (see note S2 in Rubin‐Blum et al., 2019). We found genes involved in the glycogen synthesis for carbon storage (Glg) (Sorgo et al., 2002) and cyanophycin synthesis for carbon and nitrogen storage (Cph) (Gardebrecht et al., 2012). Genes for the oxidative direction of the TCA cycle are present as well (TcaC) (Markert et al., 2007). The presence of the gene cluster of *ccoNOQP* is indicative of cytochrome cbb_3_ based oxygen respiration, able to function at low oxygen concentrations (CytCBB3) (Pitcher & Watmough, 2004). Similarly, we found the *petABC* gene cluster for cytochrome bc_1_ complex mediated electron transport (Pet) (Trumpower, 1990).

[NiFe] hydrogenase (Hup) genes were also identified in the closed genome, pointing to hydrogen oxidation capability. Recently, however, incubation experiments did not support this hypothesis (Mitchell et al., 2019). The closed genome harbours the genes of four main nitrogen processes: (1) denitrification (nitrate reduction to nitrite either performed in the periplasm by NapAB or in the cytoplasm by NarGHI, followed by the successive reductions until dinitrogen by NirS, NorCB and NosZ symbolized together here as Nirors (Gardebrecht et al., 2012; Zumft, 1997); (2) dissimilatory nitrate reduction (reduction to nitrate by NapAB or by NarGHI and electron transfer though the NADH ubiquinone oxidoreductase chain (Gardebrecht et al., 2012); (3) assimilatory nitrate reduction (symbolized here as AsN) (Girguis et al., 2000; Moreno‐Vivián et al., 1999); and, (4) an incomplete urea cycle (iUcy) lacking the last step gene for arginase. While genes encoding for ammonium transporter (AmtB), nitrate transporter (Nrt), and nitrate‐nitrite antiporter (NarK) (Gardebrecht et al., 2012), were found, four of the five urea transporter subunits‐encoding genes were absent. Consequently, uptake of urea waste from the host as previously hypothesized by Robidart et al. (2008), does not seem possible (de Oliveira et al., 2021).

We found that Endoriftia is more versatile regarding sulphur metabolism than previously reported in *Riftia‐*, and *Tevnia*‐endosymbiont associated studies (Gardebrecht et al., 2012; Kleiner et al., 2012; Robidart et al., 2008). Genes for two different mechanisms of sulphide oxidation to elemental sulphur are present: flavocytochrome c sulphide dehydrogenase (Fcsd) (Kleiner et al., 2012; Sorokin et al., 1998), and sulphide:quinone oxidoreductase (Sqr) (Dahl, 2017; Kleiner et al., 2012). Genes encoding for sulphur globule proteins, previously confirmed by electron energy loss spectrography (Pflugfelder et al., 2005), are also present in the genome (Sgp) (Dahl, 2017). Elemental sulphur from these globules could be mobilized back through the action of a polysulphide reductase (Psr; newly reported here), for sulphide supply (Robidart et al., 2008). Same function can be performed by a sulfhydrogenase in the cytoplasm (Hyd; newly reported here) (Ng et al., 2000). Additionally, both elemental sulphur and sulphide can undergo a reverse dissimilatory sulphate reductase mediated oxidation to sulphite in the cytoplasm (rDsr) (Dahl, 2017; Gardebrecht et al., 2012; Kleiner et al., 2012; Robidart et al., 2008). The resulting cytoplasmic sulphite can finally be oxidized to sulphate either by a membrane‐bound quinone‐interacting oxidoreductase (Qmo) (Dahl, 2017; Kleiner et al., 2012), by another sulphite‐oxidizing enzyme (Soe; newly reported here) (Dahl, 2017), or by the combined action of sulphate adenylyltransferase and adenylylsulphate reductase (AprSat) (Dahl, 2017; Gardebrecht et al., 2012; Robidart et al., 2008). Genes encoding for a permease and a transporter are identified in the genome to export sulphate from the cytoplasm (SulP and CysZ) (Hryniewicz et al., 1990; Zolotarev et al., 2008). Furthermore, a gene encoding for a sulphite transporter is also detected (*TauE*/*SafE*). Genes for a truncated Sox pathway are also present (tSox) (Welte et al., 2009) oxidizing thiosulphate to elemental sulphur and to sulphite, in the periplasm. The presence of putative sulphane sulphurtransferase genes (Tst; newly reported here) (Aird et al., 1987) can indicate thiosulphate disproportionation to sulphite and sulphide in the periplasm. A gene encoding for the recently characterized thiosulphate uptake protein YeeE (Tanaka et al., 2020) was also identified in the genome. Whether thiosulphate then is used for cysteine synthesis as described (Tanaka et al., 2020) needs to be further investigated.

The presence of five sets of TRAP transporter (TAXI and Dct) subunits‐encoding genes and tripartite tricarboxylate transporter Tct family subunits‐encoding genes in the genome supports the idea of organic acid internalization in the symbiont cells (Kleiner et al., 2012). *Riftia pachyptila*'s circulating fluids and tissues contain high concentration of nonessential amino acids like alanine, glutamate, glycine and serine (Cian et al., 2000). The identification of sodium/glutamate symporter‐, sodium‐glycine/alanine symporter‐, sodium/proline symporter‐ and SLC6 subfamily, as well as acetate permease‐, citrate permease‐, OprB family carbohydrate‐selective porin‐, PEP: sugar phosphotransferase system subunits‐, and oligopeptide ABC transporter‐encoding genes, suggest the possibility of amino acid transportation and independent ways of organic compound acquisition other than CO_2_ fixation in Endoriftia. Genes for three phosphate transporters were found: a low affinity transporter (Pit), a high affinity transporter (Pst), and a high affinity sodium dependent symporter (here symbolized as Hnp). Finally, we have identified the presence of complete gene set related to type‐II secretion system in the closed Endoriftia genome (Figure 3c) (Perez & Juniper, 2016), and confirmed the presence of the type IV pilus and flagellum complete gene sets (Gardebrecht et al., 2012).

### 3.3 Comparative genomics, phylogenetics and phylogenomics

To investigate the overall genomic similarity among the 12 different vestimentiferan endosymbionts found in cold‐seep and vent environments, we calculated with OrthoANI (Lee et al., 2016) the average nucleotide identity indexes using the aligned genomic regions present in the tubeworm endosymbiont genomes (Table S1). Pairwise comparisons of endosymbiont genomes revealed that the gammaproteobacteria phylotype “*Candidatus* Endoriftia persephone” is present in *Tevnia jerichonana, Ridgeia piscesae* and *Riftia pachyptila* (Gardebrecht et al., 2012; Perez & Juniper, 2016; Robidart et al., 2008), as revealed by OrthoANI score values greater than 97%. These results are in accordance with Perez and Juniper (2016). Considerably lower ANI values between 73% and 74% show that the seep endosymbionts of *Lamellibrachia luymesi*, *Lamellibrachia barhami*, *Paraescarpia echinospica*, *Seepiophila jonesi,* and *Escarpia spicata* indeed diverge from Endoriftia as previously shown by 16S rRNA similarity (McMullin et al., 2003; Reveillaud et al., 2018; Vrijenhoek, 2010) and more recent genomic studies (Breusing et al., 2020; Yang et al., 2020).

Phylogenomic and phylogenetic 16S analyses of vestimentiferan symbionts revealed the presence of two distinct and fully supported clades, one containing the hydrothermal vent “*Candidatus* Endoriftia persephone” and the other containing the cold seep representatives, in agreement with previous reports (Breusing et al., 2020; McMullin et al., 2003; Reveillaud et al., 2018; Vrijenhoek, 2010; Yang et al., 2020) (Figure S3). The ingroup relationships within the Endoriftia and “seep endosymbionts” clades differ in the phylogenomic and 16S trees, however, it is clear that *Ridgeia* endosymbionts form their own clade sister group to *Tevnia* and *Riftia* endosymbionts, as shown in previous studies (Chao et al., 2007; Feldman et al., 1997; McMullin et al., 2003; Perez & Juniper, 2016). Based on the phylogenomics/phylogenetics data, a paraphyly was observed with the endosymbionts of *Lamellibrachia luymesi* and *L. barhami*, indicating that at least two distinct gammaproteobacterial chemoautotrophic bacteria are able to infect the cold‐seep *Lamellibrachia* tubeworms, as previously reported (McMullin et al., 2003; Reveillaud et al., 2018). As a matter of fact, the OrthoANI score value for the *Lamellibrachia* endosymbiont pair is much lower (~92%) than the values herein reported for the different Endoriftia genomes (>97%), indicating that seep endosymbionts are more genetically divergent (Table S1).

A comparison of the genome sizes among vestimentiferan endosymbionts and free‐living relatives revealed that Endoriftia's closed genome (~3.6 Mb/3217 coding sequence genes (CDS)) is considerably smaller harbouring fewer CDS than that of *Sedimenticola thiotaurini* (~3.9 Mb/3739 CDS) and *S. selenatireducens* (~4.6 Mb/4276 CDS) (Flood et al., 2015; Louie et al., 2016). Therefore, the theoretical prediction of enlarged genome sizes related to facultative symbionts (Sachs et al., 2011) clearly does not hold true for Endoriftia. The genome size, GC content, and number of genes among the publicly available Endoriftia draft genomes, and the complete and closed genome herein described, are comparable (Table 1). The only exception is Robidart et al. (2008) draft genome, which presents an elevated number of predicted genes caused mainly by the fragmented nature of the assembly and possibly contamination. In fact, pairwise nucleotide similarity searches revealed distinct numbers of unmapped genomic segments in the fragmented drafts in relation to the closed genome. These unmapped genomic segments harbour many transposase, CRISPR‐Cas, and secretion system related genes. As previously reported, genes involved in the type IV and type VI secretion systems were identified in *Ridgeia*‐*Riftia‐Tevnia‐*associated Endoriftia (Gardebrecht et al., 2012; Perez & Juniper, 2016) (Table S1), but not in the closed genome herein described (where only the type II was identified). Protein secretion is key to modulate the interaction between bacteria and their environment (Tseng et al., 2009) and type II and type VI secretion systems have been recognized in other tubeworm endosymbionts. The diversity of secretion systems in Endoriftia may indicate lineage‐specific adaptations towards the free‐living and host‐associated life stages in the different populations.

**FIGURE 5:**
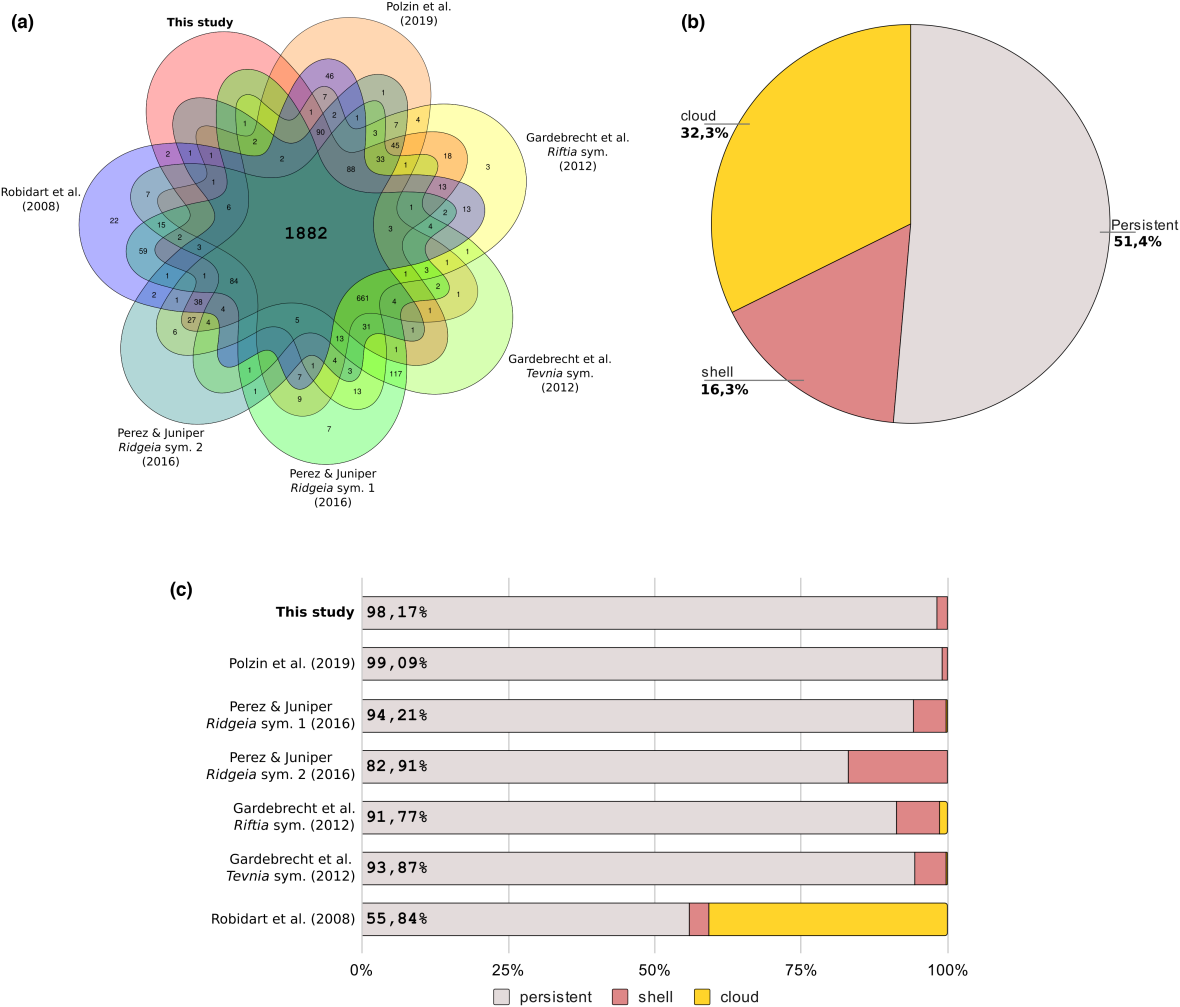
Venn diagram and pangenome of *Candidatus* Endoriftia persephone based on six publicly available drafts and the closed genome. (a) Seven set Venn diagram showing the shared orthologous groups among the six publicly available Endoriftia draft genomes and the closed genome herein described. (b) Pangenome of *Candidatus* Endoriftia persephone highlighting the persistent, shell, and cloud gene partitions. (c) Relative number of present in the three different partitions in the seven Endoriftia genomes

Orthology inferences performed with the distinct Endoriftia genomes revealed the presence of 1882 orthogroups shared by all the seven genomes, from which 1544 (~82%) were composed of single‐copy genes (Figure 5a; Table S1). Only a small number of species‐specific orthogroups were identified (i.e., paralogous groups; 32 groups), reinforcing the idea of homogeneity between the different Endoriftia populations (Gardebrecht et al., 2012). The complete and closed genome herein described, as well as the drafts obtained from Polzin et al. (2019) and Gardebrecht et al. (2012), did not contain any lineage‐specific paralogous groups. Furthermore, only 18 putative genes present in the complete Endoriftia genome, mostly composed of short repetitive sequences without any sequence similarity against the NCBI nr or eggnog databases, were not assigned to any group.

### 3.4 Pangenome of *Ca.* Endoriftia persephone

Since horizontal gene transfer events are pervasive in bacteria, the gene toolkit of an individual bacterial strain or subpopulations is much more restrained than the genetic repertoire of a species (Medini et al., 2005; Mira et al., 2010). The pangenome of Endoriftia based on the publicly available draft genomes and the closed genome (Gardebrecht et al., 2012; Perez & Juniper, 2016; Polzin et al., 2019; Robidart et al., 2008) presents a conserved core (i.e., persistent) represented by ~51% of all endosymbiont orthogroups (3048 genes) (Figure 5b). The shell and cloud orthogroup sets (noncore orthogroups present in more than one endosymbiont genome or lineage‐specific genes, respectively), comprise 16.3% (966) and 32.3% (1917) of the total pangenome (Figure 5c). Compared to the previous estimates of Perez and Juniper (2016), the Endoriftia pangenome described herein presents a higher proportion of shell and cloud genes. Only a small part of the closed Endoriftia genome (1.8%), as well as the draft genome obtained from Polzin et al. (2019) (0.9%), is composed of more phyletically restricted genes. Most of the shell genes belongs to the *Ridgeia piscesae* endosymbionts, whereas the cloud results certainly reflect the overall low quality of the initial Endoriftia draft genome assemblies (>96% of the cloud was unique to Robidart et al. (2008) draft genome; Figure 5c) rather than *bona fide* lineage‐specific genes acquired by horizontal gene transfer. Overall, our results indicate that the relatively large size of the variable genomic regions is due to sequencing and assembling biases rather than true biological variability among the different Endoriftia population, as previously discussed by Perez and Juniper (2016).

### 3.5 Mobilome of *Ca.* Endoriftia persephone

**FIGURE 6:**
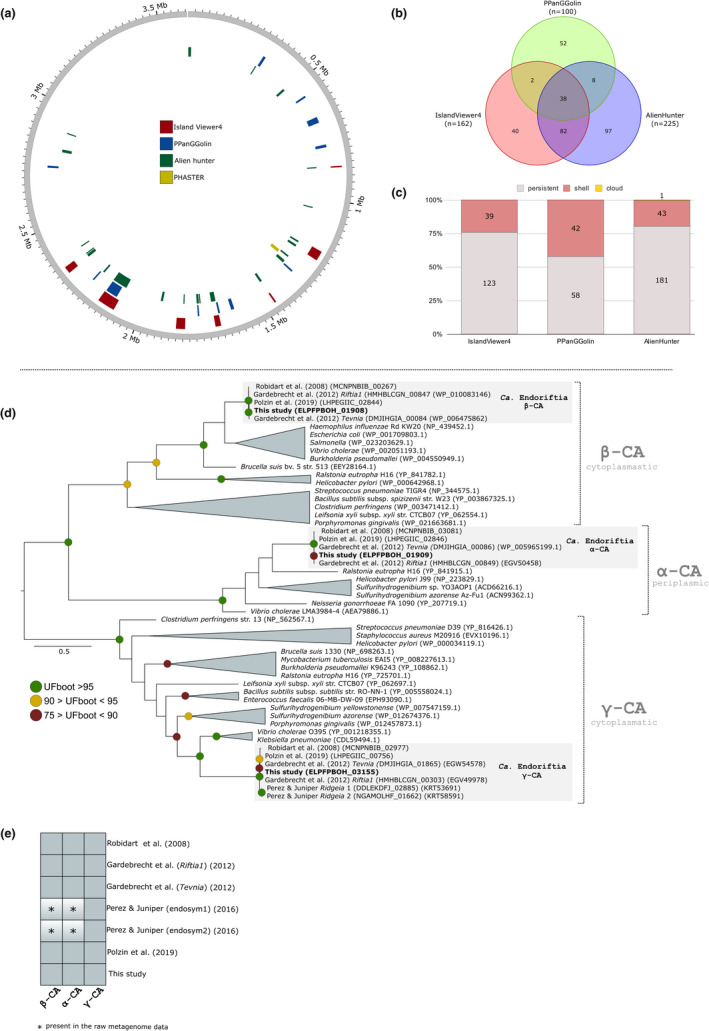
The mobilome of *Candidatus* Endoriftia persephone. (a) Genomic islands, and prophage regions identified in the closed Endoriftia genome. (b) Venn diagram depicting the overlapping genomic regions identified in the closed genome using IslandViewer4, alien_hunter and PPanGGOLin. (c) Number of persistent, shell and cloud genes located in the predicted genomic islands. (d) Maximum‐likelihood phylogenetic tree of carbonic anhydrase genes using 1000 ultrafast bootstrap replicates. Internal (generated by our annotation pipeline) and external (NCBI accession numbers) Endoriftia proteins ids are displayed in the leaves of the phylogenetic tree. (e) Distribution of carbonic anhydrase genes in the closed Endoriftia genome and in the five publicly available draft genomes. Note that despite we did not identify the genes encoding for α‐ and β‐CA in the *Ridgeia* draft genomes, these genes are indeed present in the raw metagenomic assemblies publicly available at JGI's IMG‐Mer database (GOLD analysis ID project: Ga0041824; Draft11_123875, Draft11_123872)

The acquisition of mobile genetic elements (MGEs) is known to contribute to the evolution of novel traits in bacteria (Ochman et al., 2000). MGEs are important drivers of the genomic plasticity in bacteria and could potentially facilitate host/symbiont interactions (Dietel et al., 2018; Finan, 2002; Newton & Bordenstein, 2011; Ochman & Moran, 2001; Siguier et al., 2014). Here, we fully characterize the mobilome in the closed Endoriftia genome (Figure 6).

We identified signs of a phage infection in the closed Endoriftia genome containing an incomplete ~15.2 kb prophage region that harbours seven phage‐like protein interspersed by 11 hypothetical genes without any know function (Figures 6a, S4). Sensitive taxonomic identification of the prophage region pointed to a Caudovirales infection, the most abundant phage order found at hydrothermal vents (Castelán‐Sánchez et al., 2019). Therefore, the presence of a single small and incomplete prophage sequence in the closed Endoriftia genome is not surprising, since facultative endosymbiotic bacteria are directly exposed to bacteriophages in both free‐living and intracellular environments (Metcalf & Bordenstein, 2012). It is important to stress, however, that low gene density regions containing transposases are present in the closed Endoriftia genome (as well as other Endoriftia genome drafts), which could be an indication of ancient bacteria‐phage interactions that evaded software recognition due to sequence erosion. Moreover, we screened and annotated 77 insertion sequences (IS) belonging to known described 31 different IS classes (e.g. ISXac2, ISPsy43; Table S1), hitherto uncharacterised in the Endoriftia genome. Few of these genes possibly encode for functional proteins due to the presence of integrase, core‐, and toprim‐ catalytic domains sequences. The great majority of transposons (mainly DDE‐, HTH‐ classes) found in the closed Endoriftia genome are composed of truncated and degenerated sequences indicative of a lack of complete functional transposases. A comparative study performed with 384 bacterial genomes indicated that ~2.5% of total gene number in facultative intracellular bacteria corresponded to transposon elements (Newton & Bordenstein, 2011). Interestingly, Endoriftia does not harbour as high number transposon‐encoding sequences in its genome (57 genes/~1.73% of the total number of genes) (Table S1) as would be expected to occur in facultative intracellular endosymbionts (Metcalf & Bordenstein, 2012; Newton & Bordenstein, 2011).

Despite a previous characterization of a putative plasmid containing F‐type conjugative proteins (*tra* locus) in *Riftia* 1 endosymbiont genome draft (Gardebrecht et al., 2012), the closed Endoriftia genome, as well as the remaining five investigated draft genomes, do not possess any *tra‐*related proteins. It is known that *tra* genes encode a transfer‐apparatus that aids conjugative transfer of mobile genetic elements between bacteria, despite the high energetic demand to maintain such system in a cell (Zatyka & Thomas, 1998). This finding calls for questioning this mechanism concerning the possible horizontal gene transfers among the different populations of Endoriftia. Additionally, we did not identify any extrachromosomal elements in our long‐read assembly, as reported in other studies (Gardebrecht et al., 2012; Nelson et al., 1984; Robidart et al., 2008). Finally, we found a gene that encodes a putative reverse transcriptase in the closed Endoriftia genome, which raises the possibility of foreign DNA integration into the bacterial genome.

To complement our mobilome screenings and enrich the catalogue of putative horizontally acquired genes in the closed Endoriftia genome, we employed three different methods of detecting genomic islands (i.e., large genomic regions arisen by horizontal gene transfer) (Langille et al., 2010) (Figure 6a). The number of putative horizontally acquired genomic clusters in the closed genome varies between seven (IslandViewer4) and 22 (alien hunter), ranging from 100 to 225 coding sequence genes (Table S1). Since it is well‐described in the literature that the accuracy of different genomic island prediction methods greatly varies (da Silva Filho et al., 2018; Langille et al., 2010; Lu & Leong, 2016), and many nonoverlapping predictions (Figures 6b,c) were obtained, we focused our discussion on the results supported by more than one method.

We did not identify any lineage‐specific genes (i.e., cloud genes) located in the genomic islands of the closed genome. In fact, the genes located in the genomic islands are present in nearly all six Endoriftia draft genomes, indicating that the acquisition of this putative foreign DNA predates the geographical dispersal of the different Endoriftia populations. Genes encoded in the islands contain transposition machinery, electron carrier‐, transporter, nutrition‐related, signal transduction, stress, and toxin combating, membrane‐associated, DNA modification, regulatory, and sulphur metabolism protein‐encoding genes. Nearly 45% of the predicted genes present on the genomic islands could not be assigned to any functional category and remain hypothetical in nature (Table S1).

Among these genes, we can highlight the presence of an alternative sigma factor (*algU*), which is involved in the integration of the horizontally acquired genes into the regulatory network of the bacterium host (Huang et al., 2012; Panyukov & Ozoline, 2013), and two copies of carbonic anhydrase (CA): β‐CA cytoplasmic –and α‐CA periplasmic. We identified a third carbonic anhydrase gene in the persistent genome of Endoriftia, which codes for a γ‐CA type. These three known classes are important catalysts for CO_2_ hydration reaction in bacteria responsible for not only regulating the intracellular pH (Supuran & Capasso, 2017), but also for the carbon fixation through the Calvin‐Benson cycle. They do not present any transmembrane domains and only the α‐CA harbours a signal peptide, based upon TMHMM2 and signalP‐5.0 predictions, indicating a periplasmatic location. The discovery, sequence characterization and phylogeny of CA genes in Endoriftia genome answers a long‐standing hypothesis (De Cian et al., 2003) (Figure 6d), which suggested an orchestration of cytosolic and membrane‐bound CA genes in the endosymbiont to efficiently incorporate CO_2_ from the external bacteriocyte into the bacterial cytoplasm. Our data clearly shows that Endoriftia lacks membrane‐associated CA genes to enhance CO_2_ diffusion across the cell membrane. Surprisingly, we could not locate the genes encoding for α‐ and β‐CA on the genomic islands present in the *Ridgeia* draft genomes (Perez & Juniper, 2016) (Figures 6d, S5). However, due the important role of these genes in the host‐associated and free‐living environments, this absence is probably linked to assembly biases and completeness, rather than secondary losses in this specific Endoriftia population (see Figure 6 legend for more details).

### 3.6 Absence of DNA methylation in host associated Endoriftia

Single molecule real time sequencing (SMRT) also provides information on the presence or absence of methyl modification of the genome at adenine or cytosine sites. In fact, DNA methylation is a process involved in a several biological processes in prokaryotes including regulation of DNA replication, swift responses towards environmental stresses, and host‐symbiont interactions (Casadesús & Low, 2006). Here, we investigated the base modification signals corresponding to the m4C and m6A DNA methylation in the closed Endoriftia genome. The modification quality value (QV) data indicated very low values and none of the conserved methylation motifs were detected, which points towards the absence of methylation in the host associated closed Endoriftia genome during our sampling conditions (Figure S6). However, we cannot completely rule out other symbiont subpopulations found in alternative sources (e.g. different parts of the trophosome, symbionts at the free‐living stage) that may contain signs of methylation in their genome.

Additionally, we identified in the closed Endoriftia genome an operon containing three contiguous and fragmented N‐6 adenine‐specific methyltransferase gene copies with different levels of N6_Mtase domain conservation (revealed by hmmsearch analysis against PF02384 model). Neighbouring these three genes is the presence of a gene fragment containing an inactive DDE‐type transposase suggesting a phage‐mediated gene inactivation. Similarly, bacteria possess restriction‐modification (R‐M) system to reduce the chance of invading phages (Murray, 2000). Functional type I restriction modification (RM) system comprises three subunits: HsdR, HsdM and HsdS (Murray, 2000). Interestingly, the *hsdS* gene is missing from the closed Endoriftia genome, and the RM system appears to be dysfunctional due the fragmentation of one *hsdM* and two *hsdR* genes. Furthermore, a genomic island of Endoriftia contains a putative 5‐methylcytosine‐specific restriction endonuclease McrA‐ and DNA‐cytosine methyltransferase Dcm‐encoding genes, among which the amino acid sequence of *dcm* gene could not conclusively reveal presence of any active site according to PFAM searches. Overall, the absence of methylation signals indicates that the partial methyltransferase genes are not active in the closed Endoriftia genome described herein due a series of phage transposons insertions. These results hint towards a strong dependency of Endoriftia upon CRISPR‐Cas machinery to cope with viral infections.

## 4 CONCLUSION

This study reports the closed genome of the facultative generalist *Candidatus* Endoriftia persephone from deep‐sea hydrothermal vents. We explored the closed Endoriftia genome to fully characterize the metabolic potential of *Riftia*'s endosymbiont based in a broad comparative approach focused on functional traits. This methodology enabled us to complement and link previous genomic/proteomic studies improving our understanding of Endoriftia's physiology and evolution. Additionally, due the deep long‐read sequencing we were able to uncover the full genomic content of Endoriftia allowing the correct identification of many hitherto unknown structural variations and repetitive elements, which will be a valuable resource for genetic diversity studies of uncultured bacterial taxa. The closed Endoriftia genome also enabled us to identify and annotate genomic regions linked to horizontally transferred events and to analyse the epigenetic landscape in this bacterium, raising interesting questions about the evolution of the methylome and mobilome in facultative endosymbiotic systems. To conclude, the Endoriftia genome is the first closed endosymbiont genome obtained from a vestimentiferan tubeworm and will be valuable for a plethora of comparative physiological and evolutionary studies within the vestimentiferan‐microbe mutualism field, as well as many in other metazoan‐symbiont systems.

## AUTHOR CONTRIBUTIONS

M.B. collected the sample, A.L.de.O. purified and extracted the Endoriftia DNA, developed the bioinformatic pipeline, executed all in silico analyses, and wrote the initial draft of the study. S.E.‐H. and A.S. manually curated the in silico results. S.E.‐H., M.B. and A.S. conceptualized and performed the trait analyses described in this manuscript. All authors commented and approved the final version of this manuscript.

## CONFLICT OF INTEREST

The authors declare no conflict of interest.

### OPEN RESEARCH BADGES

This article has earned an Open Data badge for making publicly available the digitally‐shareable data necessary to reproduce the reported results. The data is available under the BioProject PRJNA762254.

## Supporting information


**Figure S1** Gene ontology (GO) enrichment analyses for the positively selected genes identified in the complete and closed Endoriftia genome.
**Figure S2** Maximum parsinomy (left) and maximum likelihood gene tree (right) reconstructions with several gene orthologs of *aclB* and *sucC*.
**Figure S3** Rooted maximum‐likelihood phylogenomic (left) / phylogenetic 16S (right) trees of tubeworm endosymbionts using 1000 rapid bootstrap and SH‐aLTR test replicates.
**Figure S4** Prophage region identified in the complete and closed Endoriftia genome F.
**Figure S5** Genomic islands identified by IslandViewer4 and their distribution in the seven Endoriftia genomic datasets.
**Figure S6** Modification quality value (QV) and scatter plots of sequencing coverage with the identified motif sites.

**Table S1** Supplementary information associated with all the analyses performed in this manuscript.

**Table S2** Manually curated list of traits and their associated genes.

**Data S1** Zip file containing annotation/intermediary files, as well as in‐house bioinformatic scripts used in this manuscript.

## Data Availability

The raw long‐read sequencing data (accession no. SRR15838435) and the complete closed genome (accession no. CP090569) have been deposited in the SRA and GenBank databases under the BioProject PRJNA762254. The CRISPR spacers found in the closed Endoriftia genome, the protein coding sequences, and annotation files generated for the closed Endoriftia genome, as well as intermediary files and in‐house bioinformatic scripts used in this manuscript are available in the Data S1.
