# Supplementary material for: The complete and closed genome of the facultative generalist Candidatus Endoriftia persephone from deep‐sea hydrothermal vents
Source: Mol Ecol Resour. 2022 Jun 30;22(8):3106–23. doi: 10.1111/1755-0998.13668 (PMC9796809; doi:10.1111/1755-0998.13668)
Supplement: Supplementary file 1 — Figure S1 Gene ontology (GO) enrichment analyses for the positively selected genes identified in the complete and closed Endoriftia genome. Figure S2 Maximum parsinomy (left) and maximum likelihood gene tree (right) reconstructions with several gene orthologs of aclB and sucC. Figure S3 Rooted maximum‐likelihood phylogenomic (left) / phylogenetic 16S (right) trees of tubeworm endosymbionts using 1000 rapid bootstrap and SH‐aLTR test replicates. Figure S4 Prophage region identified in the complete and closed Endoriftia genome F. Figure S5 Genomic islands identified by IslandViewer4 and their distribution in the seven Endoriftia genomic datasets. Figure S6 Modification quality value (QV) and scatter plots of sequencing coverage with the identified motif sites. [file MEN-22-3106-s001.docx]

**The complete genome of the facultative generalist *Candidatus* Endoriftia persephone from deep-sea hydrothermal vents**

André Luiz de Oliveira^1^, Abhishek Srivastava^1^, Salvador Espada-Hinojosa^1^, Monika Bright^1^

^1^ Department of Functional and Evolutionary Ecology, University of Vienna, Djerassiplatz 1, Vienna, Austria.

Corresponding authors: André Luiz de Oliveira ([andre.luiz.de.oliveira@univie.ac.at](mailto:andre.luiz.de.oliveira@univie.ac.at)) and Monika Bright (monika.bright@univie.ac.at)


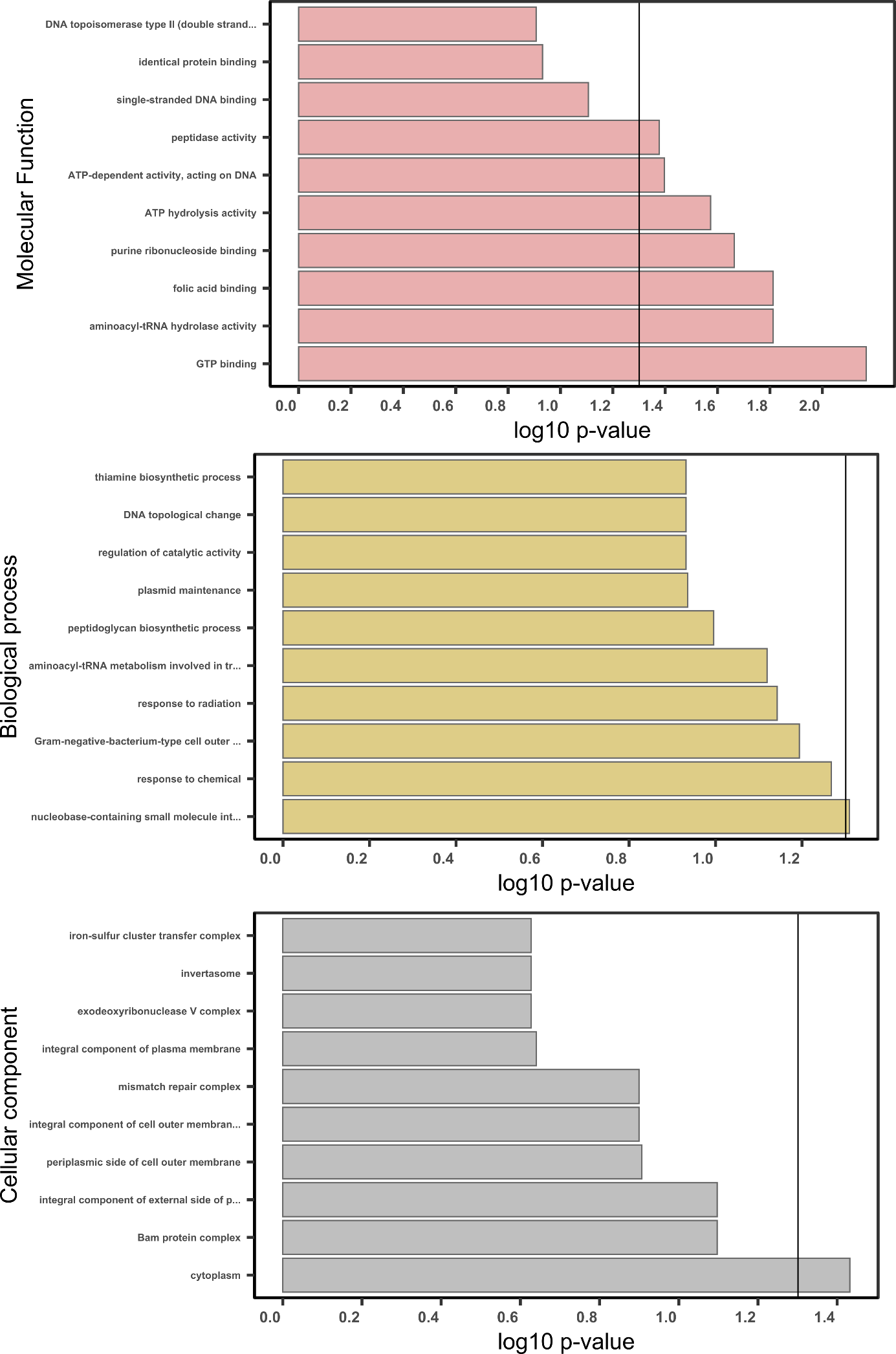


**Supplementary Figure 1 - Gene ontology (GO) enrichment analyses for the positively selected genes identified in the complete and closed Endoriftia genome.** The graphs correspond to three domains of ontologies: molecular function (MF), biological process (BP), and cellular component (CC). The selected genes were analysed for enrichment in specific GO categories using the TopGO program against the background (all coding sequence genes). Y axis corresponds to enriched GO terms found in the respective domains (BP, MF and CC). X axis correspond to the log function of Fisher p-values obtained for each one of the enriched terms. The back line denotes a p-value = 0.05. P-values greater than 1,30 (log 0,05) indicate statistically significant enriched term.

**
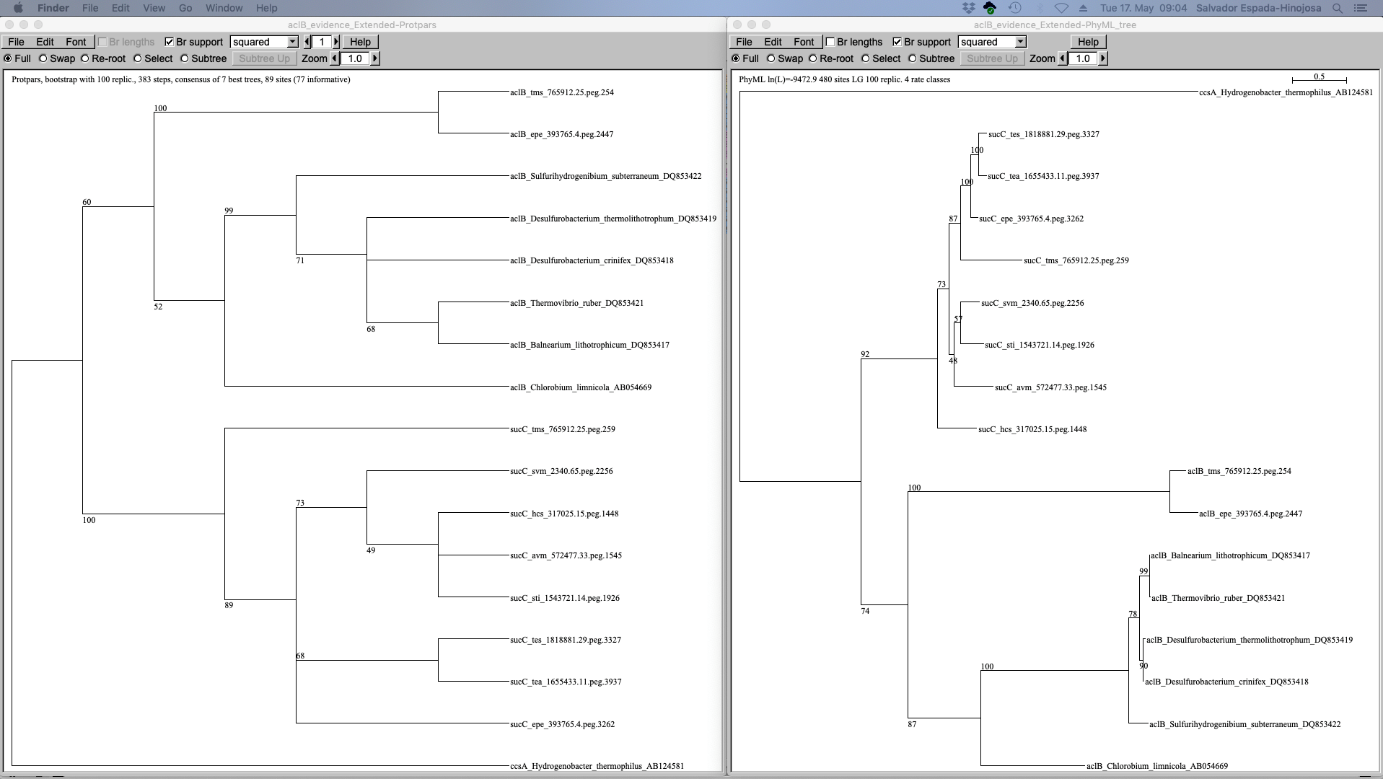
Supplementary Figure 2 - Maximum parsimony (left) and maximum likelihood gene tree (right) reconstructions with several gene orthologs of *aclB* and *sucC*.** The gene trees corroborate that the *aclB* and *sucC* Endoriftia genes cluster with their respective homologs. epe, *Candidatus* Endoriftia persephone; tes, *Candidatus* Thiodiazotropha endoloripes; tea, *Candidatus* Thiodiazotropha endolucinida; hcs, *Hydrogenovibrio* crunogenus; sti, *Sedimenticola thiotaurini*; svm, *Solemya velum*; tms, *Thioflavicoccus mobilis*. Both trees were calculated over a Muscle alignment in SeaView v4.5.4 with default parameters and 100 non-parametric bootstraps).

**
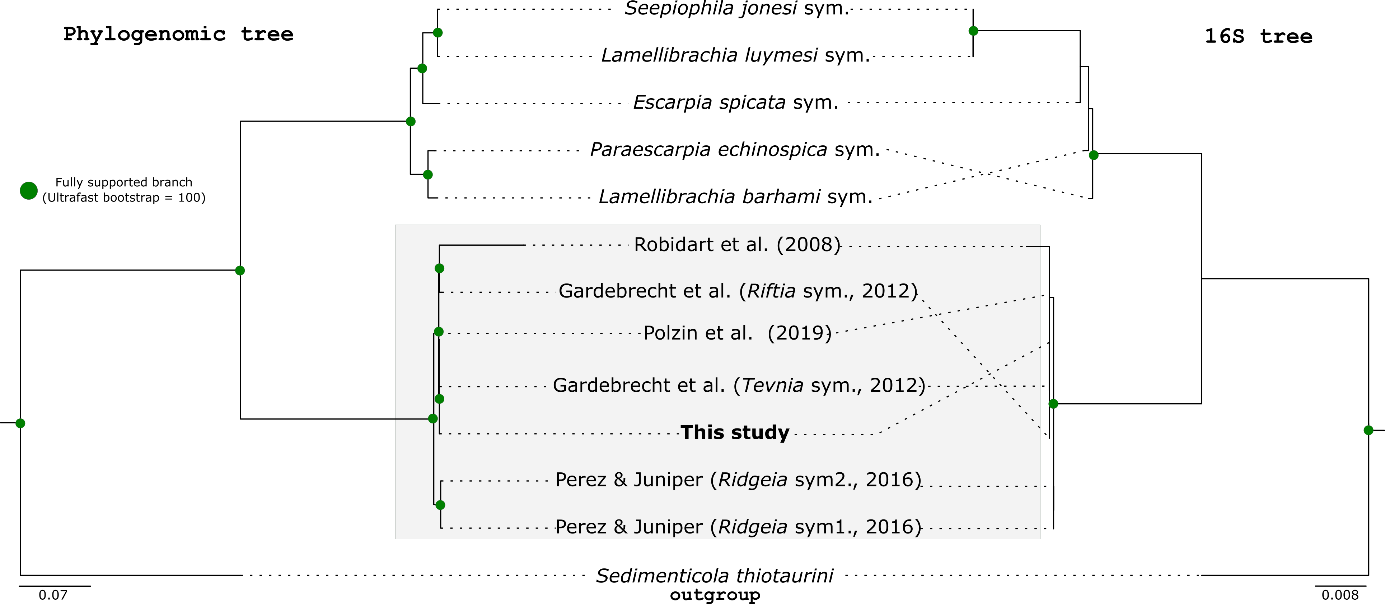
Supplementary Figure 3**- Rooted maximum-likelihood phylogenomic (left) / phylogenetic 16S (right) trees of tubeworm endosymbionts using 1000 rapid bootstrap and SH-aLRT test replicates. The supermatrix was generated concatenating 1,879 shared orthogroups. 16S sequences were obtained from the different drafts by sequence similarity searches using the reference sequences obtained from the complete and closed genome. Node supports correspond to SH-aLRT and bootstrap values. The grey box indicates the unique endosymbiont phylotype of *Tevnia*, *Ridgeia* and *Riftia* tubeworms. Fully supported nodes are indicated by green circles in the tree nodes.


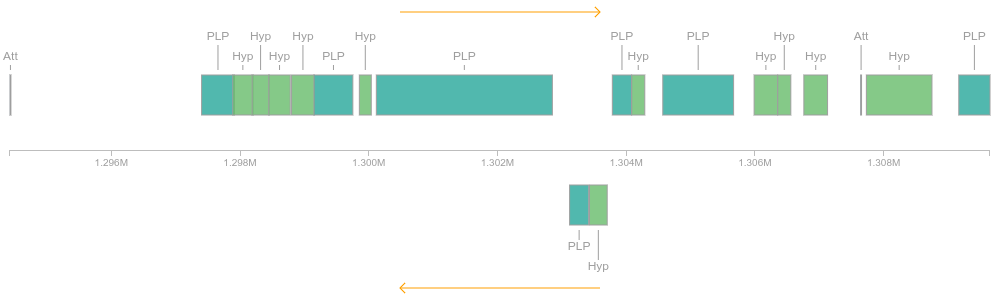


**Supplementary Figure 4 – Prophage region identified in the complete and closed Endoriftia genome.** The 15,2 kb incomplete phage region located in the closed genome of Endoriftia. Sensitive similarity searches identified a Caudovirales infection.


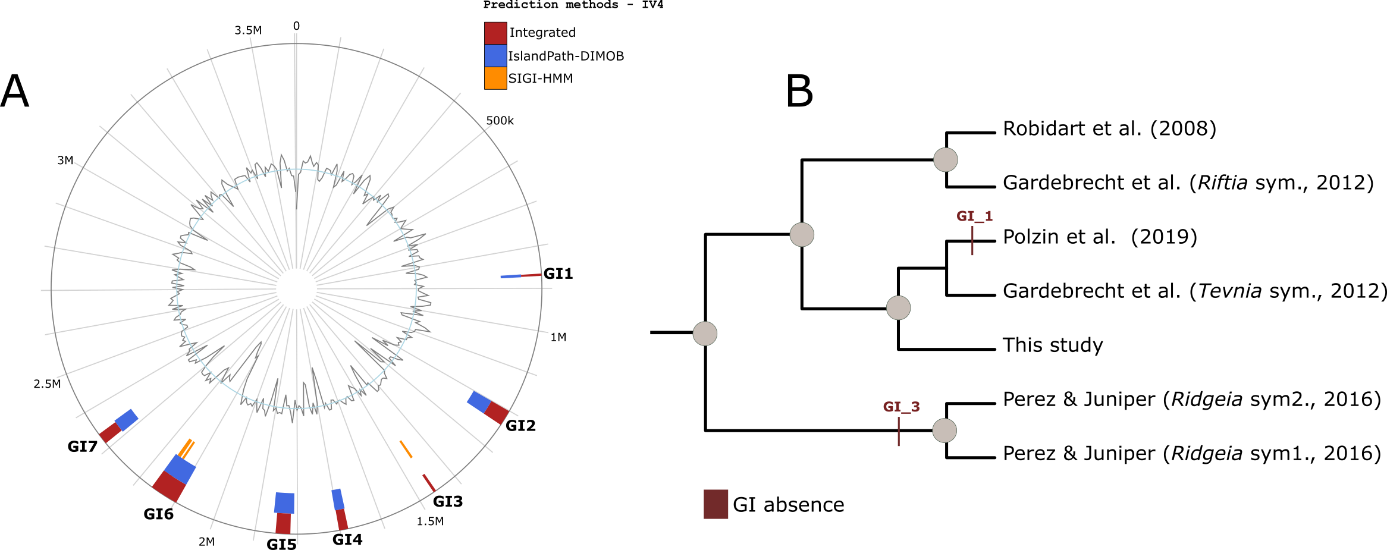


**Supplementary Figure 5 – Genomic islands identified by IslandViewer4 and their distribution in the seven Endoriftia genomic datasets. A,** IslandViewer4 identified seven genomic islands present in the complete and closed Endoriftia genome harbouring 162 coding sequences. **B,** Genomic islands 1 and 3 identified in the complete and closed genome are missing in the Polzin et al. (2019) and Perez & Juniper (2016) Endoriftia drafts.


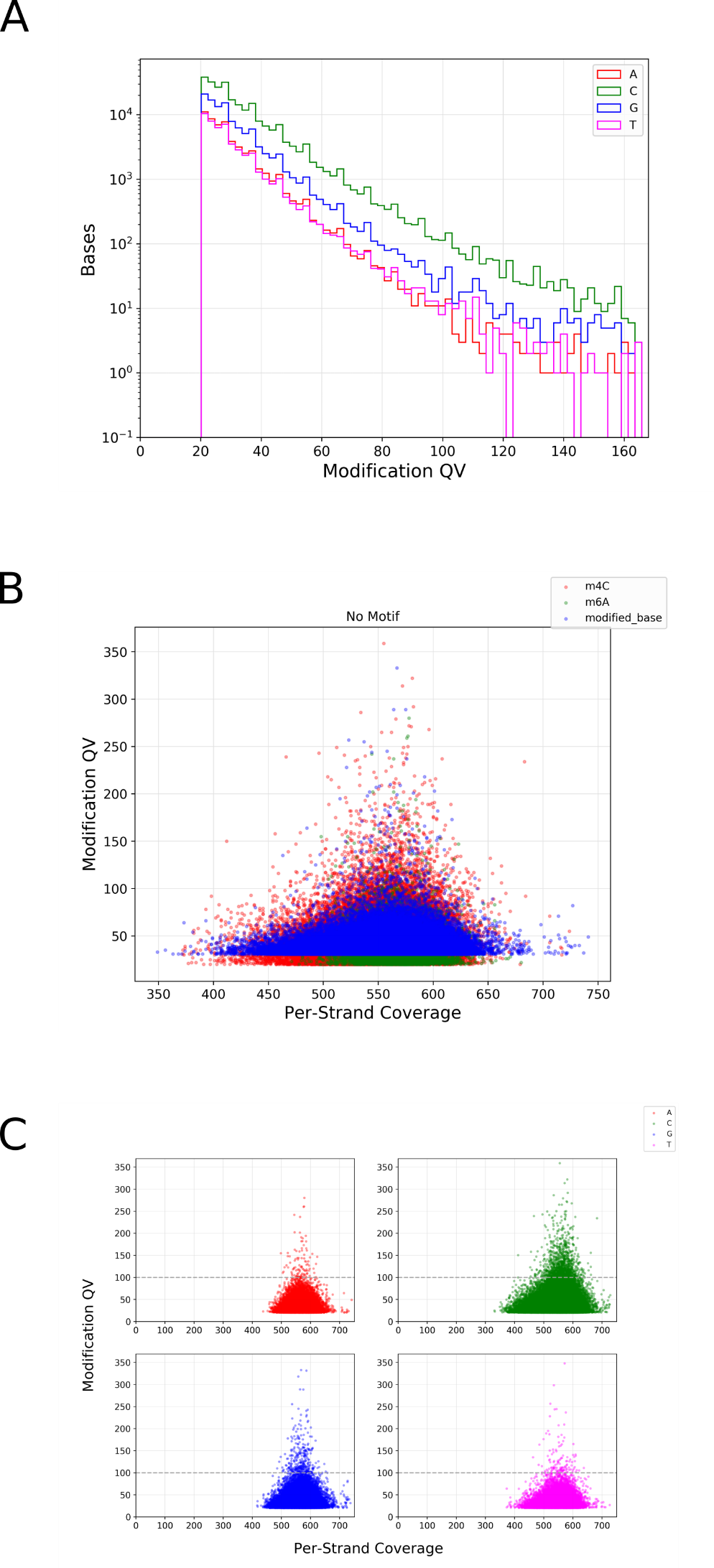


**Supplementary Figure 6** – Modification quality value (QV) and scatter plots of sequencing coverage with the identified motif sites. The low QV **(A)**, absence of conserved methylation motifs **(B-C)**, as well as the incomplete methyltransferase domains identified in important genes related to epigenetic regulation in Endoriftia (see main text) suggest a lack of methylation in the host-associated Endoriftia genome herein described.
